# Supplementary material for: Mobile class A β-lactamase gene bla GMA-1
Source: Microbiol Spectr. 2023 Dec 11;12(1):e02589-23. doi: 10.1128/spectrum.02589-23 (PMC10782965; doi:10.1128/spectrum.02589-23)
Supplement: Fig. S1 — bla GMA-1 insertion locations without SE genes. [file spectrum.02589-23-s0001.docx]

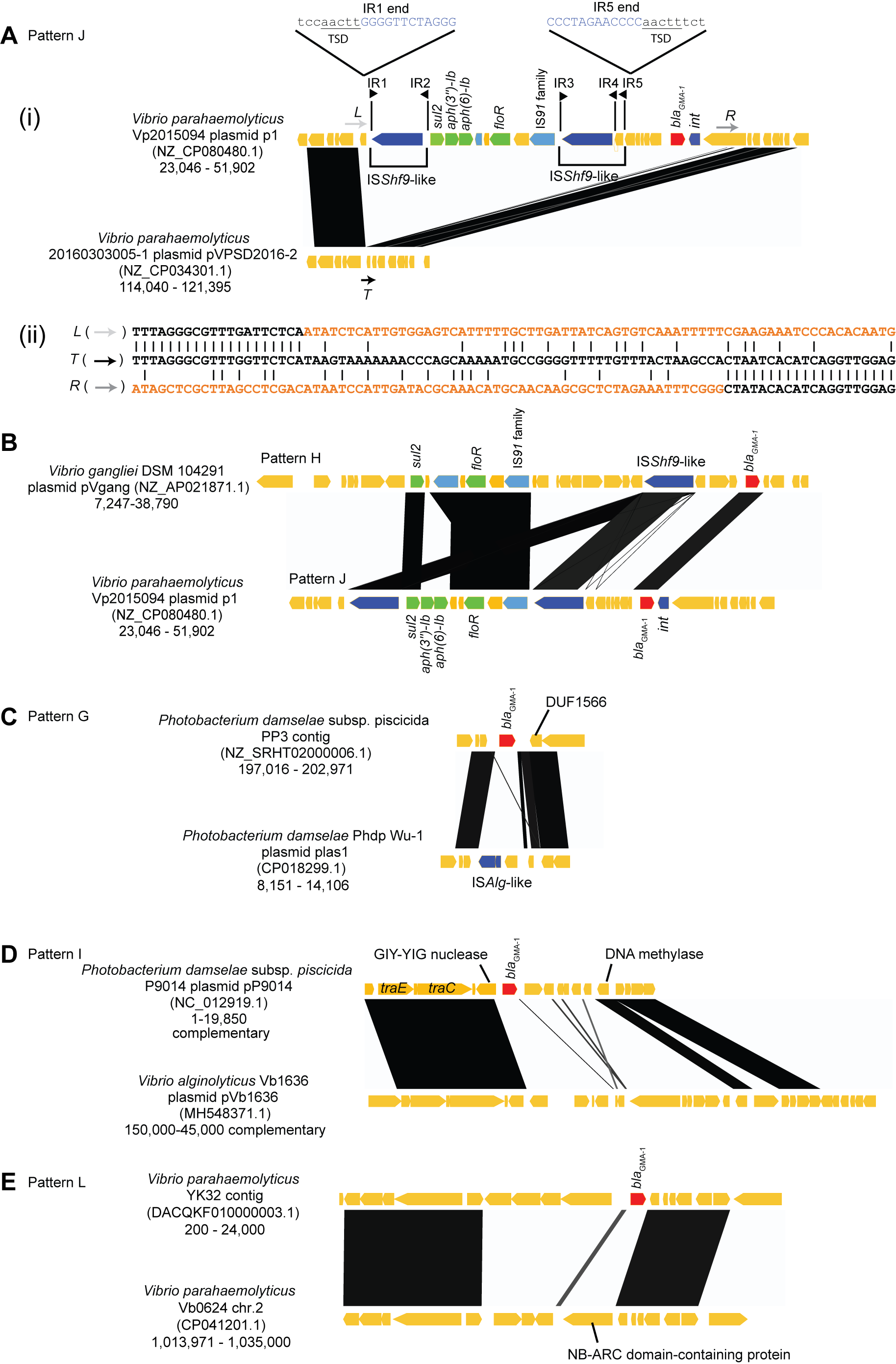


**FIG S1** *bla*_GMA-1_ insertion locations without SE genes. (A) Pattern J. The insertion contains an *int* gene near *bla*_GMA-1_. IS*Shf-9*-like elements brackets four antimicrobial resistance genes shown in green. (i) Structural comparison between p1 and pVPSD2016-2. (ii) Alignment of border regions between putative inserts and plasmid backbone of two plasmids. *L*: left border; *R*: right border; *T*, target sequence. Middle: alignment of *L*, *R*, and *T*. (B) Pattern H. pVgang has no other related sequenced plasmids in the RefSeq/GenBank database, so the plasmid backbone and insert region could not be identified. *bla*_GMA-1_ flanks contain IS*Shf-9*-like elements and other genes observed in the p1 insert. (C) Pattern G. *bla*_GMA-1_ translocation on a plasmid-like contig without an identifiable target site duplication (TSD). (D) Pattern I. *bla*_GMA-1_ insertion next to a GIY-YIG nuclease gene on pP9014. The closet plasmid has a different insertion at the equivalent location. (E) Pattern L. *bla*_GMA-1_ translocation on the YK32 chromosome without an identifiable TSD. The closet chromosome has a different insertion at the equivalent location.
